# Integrating surface‐guided radiation therapy and continuous positive airway pressure for breast cancer: Improving reproducibility and efficacy

**DOI:** 10.1002/acm2.70284

**Published:** 2025-10-09

**Authors:** Jin Dong Cho, Su Chul Han, Jason Joon Bock Lee, Hyebin Lee, Heerim Nam

**Affiliations:** ^1^ Department of Radiation Oncology Kangbuk Samsung Hospital Sungkyunkwan University School of Medicine Seoul Republic of Korea

**Keywords:** continuous positive airway pressure, displacement monitoring, surface guided radiation therapy

## Abstract

**Background:**

The utility of surface‐guided radiation therapy (SGRT) with continuous positive airway pressure (CPAP) remains underexplored compared to its application with deep inspiratory breath hold (DIBH). This study investigates the integration of CPAP and SGRT, focusing on positional reproducibility and treatment efficiency.

**Purpose:**

This study evaluated the impact of patient surface displacement during breast cancer radiation therapy using optical and thermal SGRT monitoring and compared treatment time characteristics between patients undergoing SGRT, either with or without CPAP, and a cohort of patients undergoing treatment without SGRT.

**Methods:**

The SGRT cohort comprised thirty patients: 15 with CPAP (CPAP + SGRT) and 15 without CPAP (SGRT‐only). The surface displacement was monitored using an advanced optical and thermal SGRT system with thresholds of 3 mm for translational and 2.5° for rotational displacement. Treatment workflow metrics and positional deviations were assessed across 16 fractions. A comparative analysis included a cohort of 27 free‐breathing (FB) patients who did not receive SGRT.

**Results:**

Positional reproducibility was similar in both SGRT groups, with translation vectors of 1.46 ± 0.98 mm (CPAP + SGRT) and 1.37 ± 0.80 mm (SGRT‐only) and rotation vectors of 0.57 ± 0.40° and 0.57 ± 0.39°, respectively. Despite comparable displacement control, treatment delivery time variability was highest in the CPAP + SGRT group (normalized standard deviation: 0.16), followed by the SGRT‐only (0.11) and FB groups (0.03). The broader time distributions in the SGRT group were attributed to beam‐hold activations exceeding the displacement thresholds, whereas total treatment time did not differ significantly between groups.

**Conclusions:**

SGRT effectively minimized displacement‐related uncertainties during breast cancer radiation therapy with and without CPAP. Although CPAP provides additional internal stabilization and its integration with SGRT increased treatment delivery time variability, the total treatment time remained comparable across all groups. These findings underscore the potential of SGRT and CPAP as complementary tools to enhance precision and safety, particularly for techniques requiring high positional accuracy.

## 1 INTRODUCTION

Surface‐guided radiation therapy (SGRT) has emerged as a pivotal advancement in radiation oncology, offering real‐time monitoring of patient surface displacement during treatment.^1^ By tracking the external surface of the patient, SGRT enables the precise targeting of radiation beams while minimizing exposure to adjacent normal tissues. Optical surface‐guided systems provide precise external surface tracking using advanced imaging technologies, and thermal systems complement this by offering temperature‐based verification to improve surface registration accuracy. Together, these systems enable a more comprehensive approach for monitoring and improving breast positional reproducibility during RT.^2^ A key feature of SGRT is its ability to automatically pause radiation delivery when discrepancies between the live and reference surfaces exceed predetermined thresholds, thereby enhancing treatment safety and accuracy. The integration of such real‐time monitoring tools is particularly relevant in certain areas of the body, such as the thoracic region, where movement can compromise treatment precision.^1^, ^2^, ^3^, ^4^

Managing organ movements, especially breathing, poses a critical challenge in thoracic radiation therapy. Various techniques such as continuous positive airway pressure (CPAP) have been employed to reduce respiratory amplitude. CPAP offers considerable volumetric and dosimetric benefits by stabilizing the lungs and chest walls during radiation delivery.^5^ By maintaining consistent lung inflation, CPAP reduces the risk of pulmonary and cardiac toxicities, which are common concerns in the radiation treatment of thoracic malignancies, including breast cancer. The application of CPAP in breast cancer radiation therapy, particularly in left‐sided cases where the heart is in close proximity to the target area, has demonstrated its potential to reduce the radiation dose in critical organs.^5^, ^6^, ^7^, ^8^, ^9^

The combined use of SGRT and CPAP presents an innovative approach to further enhance clinical outcomes by optimizing both the precision and safety of RT. While SGRT provides real‐time surface monitoring, CPAP helps stabilize the internal anatomy, potentially improving treatment accuracy and reducing radiation exposure to non‐target tissues. However, despite the growing interest in these techniques, research on their combined use in breast cancer radiation therapy remains limited compared to its application with deep inspiratory breath hold (DIBH).

Given the complementary roles of SGRT and CPAP in radiation therapy, we hypothesized that the positional reproducibility achieved with the combined use of CPAP + SGRT would be comparable to or not inferior to that achieved with SGRT alone. This study aimed to evaluate positional reproducibility during breast cancer radiation therapy using optical and thermal SGRT systems.

Furthermore, this study analyzed treatment time metrics—including total treatment time, treatment delivery time, and beam‐on time—to compare the treatment efficiency and temporal consistency among the CPAP + SGRT, SGRT‐only, and free‐breathing (FB) groups. By investigating both displacement control and temporal performance, this study aimed to provide a comprehensive assessment of the clinical value of integrating SGRT and CPAP for improving treatment precision without compromising workflow efficiency.

## 2 MATERIALS AND METHODS

### 2.1 SGRT monitoring system

The SGRT system used in this study was the BrainLab ExacTrac Dynamic (EXTD) system (BrainLab AG, Munich, Germany). The EXTD integrates advanced optical and thermal imaging with stereoscopic x‐ray technology to achieve precise patient positioning and real‐time displacement monitoring during radiotherapy. The system consists of an overhead optical and thermal imaging module and two stereoscopic x‐ray tubes mounted on the treatment room floor paired with flat‐panel detectors on the ceiling. This configuration provided high‐resolution positional accuracy for both external and internal surface tracking.^3^, ^10^

The optical subsystem uses structured light projection combined with stereoscopic cameras to generate a live three‐dimensional (3D) map of the patient's surface. The system captures and processes surface images at a frame rate of approximately 15–20 frames per second (FPS), enabling real‐time tracking of patient surface displacement throughout treatment. The thermal imaging module complements this by adding temperature information, which enhances positional tracking accuracy. For internal imaging, stereoscopic radiography was used to verify the alignment of the internal anatomy with the planned position. All measurements and corrections were performed within a six‐degree‐of‐freedom (6DOF) framework, including translational (lateral, longitudinal, and vertical) and rotational (pitch, roll, and yaw) displacements.

### 2.2 Patient setup and surface displacement monitoring

Prior to the treatment, a reference surface was created for each patient using body contour data from the planning computed tomography (CT) images. This reference surface was uploaded to the EXTD system and served as the baseline for patient positioning during treatment. For patients in the CPAP + SGRT group, a CPAP device was used to maintain a pressure of 15 cm H_2_O during the CT simulation. This pressure setting was consistently applied throughout the treatment to ensure reproducibility of the reference surface and positional alignment. All other patients, including those in the SGRT‐only and FB groups, underwent CT simulation under free‐breathing conditions. CPAP is a noninvasive technique that maintains positive pressure in the airways throughout the breathing cycle. In the context of radiation therapy, it facilitates consistent lung inflation, reduces internal organ motion, and improves dosimetric outcomes by displacing critical structures such as the heart and lungs away from the treatment field.^5^, ^8^, ^11^

The patients were initially positioned on the treatment couch using an EXTD optical camera system, which compared the live surface image with the reference surface. A pretreatment kV image was acquired and matched using digitally reconstructed radiographs from the planning CT. Any detected deviations were corrected by adjusting the automated treatment couch to ensure the accurate alignment in 6DOF.

Throughout the treatment, real‐time displacement monitoring was performed using EXTD optical and thermal imaging systems. The system continuously compared the live surface image with the reference surface and calculated the deviations in the translational and rotational directions. Automatic beam‐hold functionality was activated when the displacement exceeded preset thresholds of 3 mm for translations and 2.5 for rotations. Regions of interest were defined on the patient's surface, focusing on areas relevant to the treatment target. This ensured that patient displacement within the treatment field was accurately monitored and managed.^1^, ^12^, ^13^

### 2.3 Patient groups

From February 2024 to July 2024, consecutive patients with breast cancer who received radiation therapy were enrolled. Patients with left‐sided breast cancer consented to use CPAP, while those with right‐sided breast cancer were treated with SGRT alone. Volumetric modulated arc therapy (VMAT) was employed for all patients, with treatment plans generated using the Eclipse treatment planning system (Varian Medical Systems, Palo Alto, CA, USA).

This study included 30 patients with breast cancer who underwent radiotherapy using the EXTD system for displacement monitoring and positional accuracy. Patients were divided into two groups based on the treatment protocol. The first group, referred to as the CPAP + SGRT group, comprised 15 patients who underwent radiotherapy combined with CPAP and SGRT. The second group, the SGRT‐only group, included 15 patients who underwent SGRT without CPAP. Treatments in this group were delivered under free‐breathing conditions while using real‐time displacement monitoring and gating via the EXTD optical camera system. Both groups followed identical setups and monitoring procedures using the EXTD system to ensure precise treatment delivery through real‐time displacement tracking and automatic beam‐hold functionalities. The positional reproducibility of all groups was assessed by analyzing the deviations in translational and rotational displacement vectors across fractions. The variance in the positional deviations between successive treatment fractions was used as a measure of reproducibility.

In addition to these two groups, a separate cohort of 27 patients treated with the FB technique was included in the comparative analysis. The treatment time metrics among the CPAP + SGRT, SGRT‐only, and FB cohorts was assessed to evaluate the impact of the different approaches on treatment efficiency and consistency.

### 2.4 Data analysis

This study analyzed displacement data from 480 treatment fractions, obtained from 30 patients in the SGRT groups—15 in the CPAP + SGRT group and 15 in the SGRT‐only group—each receiving 16 fractions. For the evaluation of treatment time metrics, a total of 912 fractions were included, consisting of 240 fractions from the CPAP + SGRT group, 240 from the SGRT‐only group, and 432 from the FB group.

The displacement data for all patients were extracted from the EXTD system logs, capturing deviations in the translational and rotational directions. Positional shifts were recorded across three translational axes (lateral, longitudinal, and vertical) and three rotational axes (pitch, roll, and yaw). For each treatment fraction, the vector magnitudes of the translational and rotational shifts were calculated to quantify overall patient displacement.

The translational and rotational displacement vectors between the CPAP + SGRT and SGRT‐only groups were compared using the bootstrap method, which was also used to estimate the variability in displacement data. The displacement distributions were visualized using histograms, providing a detailed view of the frequency and spread of displacement across all 6DOF. To further evaluate displacement control specifically during beam‐on periods, an additional subset analysis was performed using displacement data recorded only when the patient's surface remained within the predefined gating thresholds (3 mm for translation and 2.5° for rotation). Translational and rotational deviations during these gated intervals were computed and compared between the CPAP + SGRT and SGRT‐only groups.

In this study, treatment delivery time was defined as the interval between the first beam‐on and the final beam‐off during radiation treatment. This includes all time periods when radiation delivery was temporarily paused due to displacement exceeding the gating thresholds. The beam‐on time refers exclusively to the cumulative duration in which the linear accelerator was actively delivering radiation within the gating thresholds.

To assess the impact of beam‐hold events on treatment delivery time variability, each fraction was normalized using the mean treatment delivery time for the corresponding patient. The standard deviation (Std) of these normalized values was then calculated to evaluate the consistency of beam delivery across groups, independent of absolute treatment durations.

In addition to analyzing displacement vectors and evaluating treatment delivery time, treatment efficiency was assessed by calculating the duty cycle. The duty cycle was defined as the ratio of beam‐on time—the cumulative duration during which radiation was delivered while the patient's displacement remained within the gating threshold—to the total treatment time. Total treatment time was defined as the interval between the acquisition of the first pre‐treatment image (used for initial image guidance) and the termination of the final beam. To compare duty cycles among the CPAP + SGRT, SGRT‐only, and FB Breast groups, the mean and Std were calculated. Total treatment time were also compared across the three groups using the same statistical approach. All time parameters were expressed in minutes (min) throughout the analysis. These time metrics—including treatment delivery time, beam‐on time, and total treatment time—were automatically recorded in the treatment planning system (TPS) and linear accelerator log files, ensuring objective and consistent data collection across all fractions.

Statistical analyses were conducted using the bootstrap method and Mann–Whitney U test. The bootstrap method was selected owing to its robustness in handling large datasets generated from real‐time displacement monitoring.^14^, ^15^, ^16^, ^17^ As a non‐parametric approach, it does not assume normality in data distribution and allows reliable estimation of confidence intervals and statistical significance, making it particularly well‐suited for analyzing variability in displacement data.

Given the limited number of patients in each group, the Mann–Whitney U test was used to detect differences in the treatment time metrics between the three groups.^18^, ^19^, ^20^, ^21^ Using this test, we evaluated differences in treatment time metrics and displacement patterns among the CPAP + SGRT, SGRT‐only, and FB groups, and the results provided important insights into the impact of these treatment approaches on patient care.

## 3 RESULTS

### 3.1 Translation and rotation vector analysis

**FIGURE 1:**
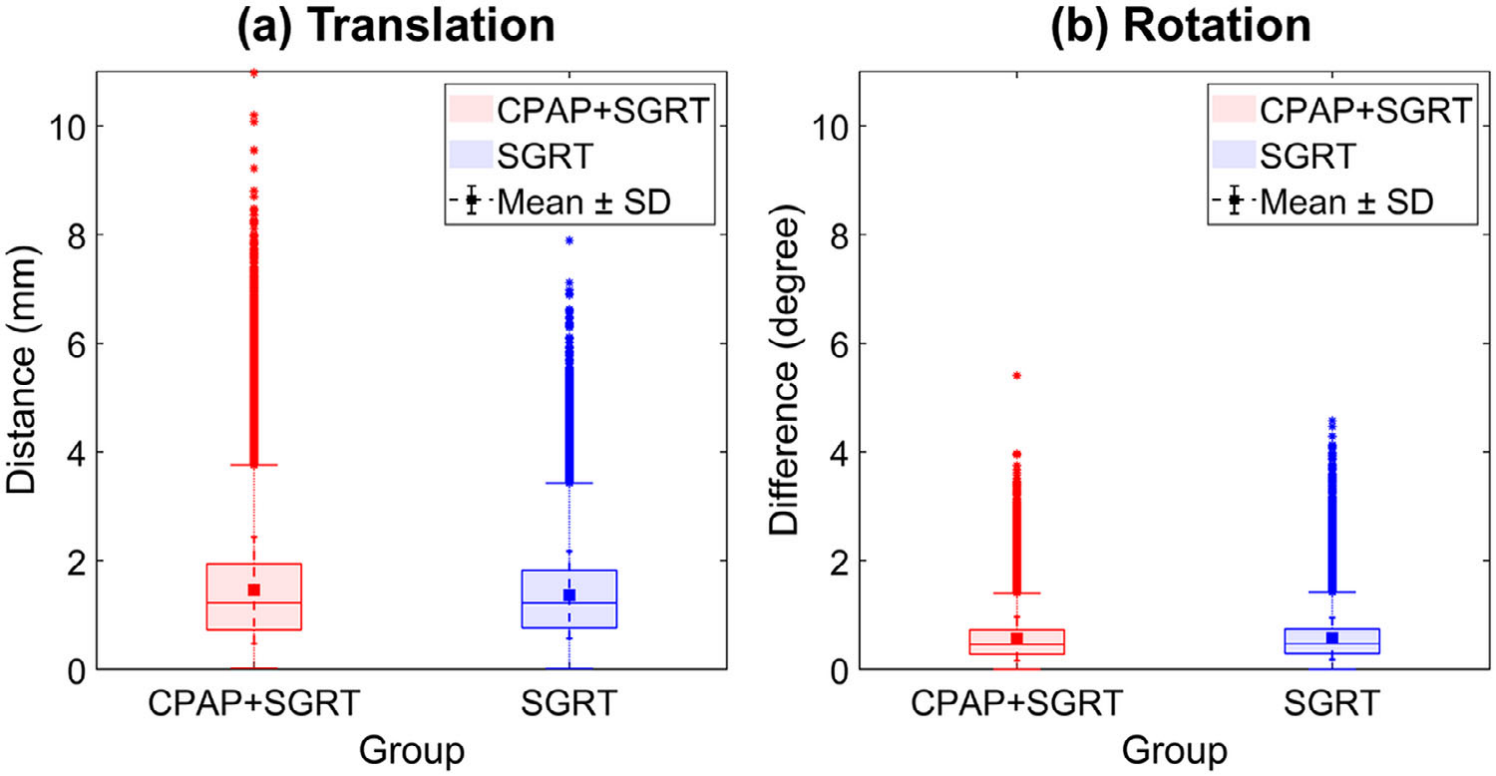
The difference in displacement between live and reference surface images was represented as (a) translation and (b) rotation vectors. A comparison was conducted between the CPAP + SGRT and SGRT‐only groups.

Analysis of patient displacement in the CPAP + SGRT and SGRT‐only groups showed minimal differences in both translation and rotation vectors. The translation vector for the CPAP + SGRT and SGRT‐only groups were 1.46 ± 0.98 mm and 1.37 ± 0.80 mm, respectively. (Figure 1, Table 1). Similarly, the rotation vector for both groups was nearly identical, with 0.57 ± 0.40° and 0.57 ± 0.39° recorded for the CPAP + SGRT SGRT‐only groups, respectively (Figure 1, Table 1).

**TABLE 1:** Statistical values for the CPAP + SGRT and SGRT‐only groups were calculated for each displacement difference and analyzed using the bootstrap method. Translation values are presented in millimeters (mm), and rotation values are presented in degrees (°). Abbreviations: In this table, Std denotes standard deviation, Max denotes maximum, and Min denotes minimum.

Displacement	Group	Mean	Median	Std	Max	Min	Bootstrap *p*‐value
**Translation**	**CPAP + SGRT**	1.46	1.22	0.98	10.98	0.02	0.49
	**SGRT**	1.37	1.22	0.80	9.84	0.01	
**Rotation**	**CPAP + SGRT**	0.57	0.46	0.40	5.41	0.00	0.49
	**SGRT**	0.57	0.48	0.39	4.58	0.00	

The most significant patient displacement recorded during treatment was a translation vector of 10.98 mm and rotation vector of 5.41 (Figure 1, Table 1). Despite these outliers, the overall displacement between the two groups did not show statistically significant differences. Bootstrap analysis confirmed the absence of statistically significant differences in patient displacement between the CPAP + SGRT and SGRT‐only groups (Table 1).

**FIGURE 2:**
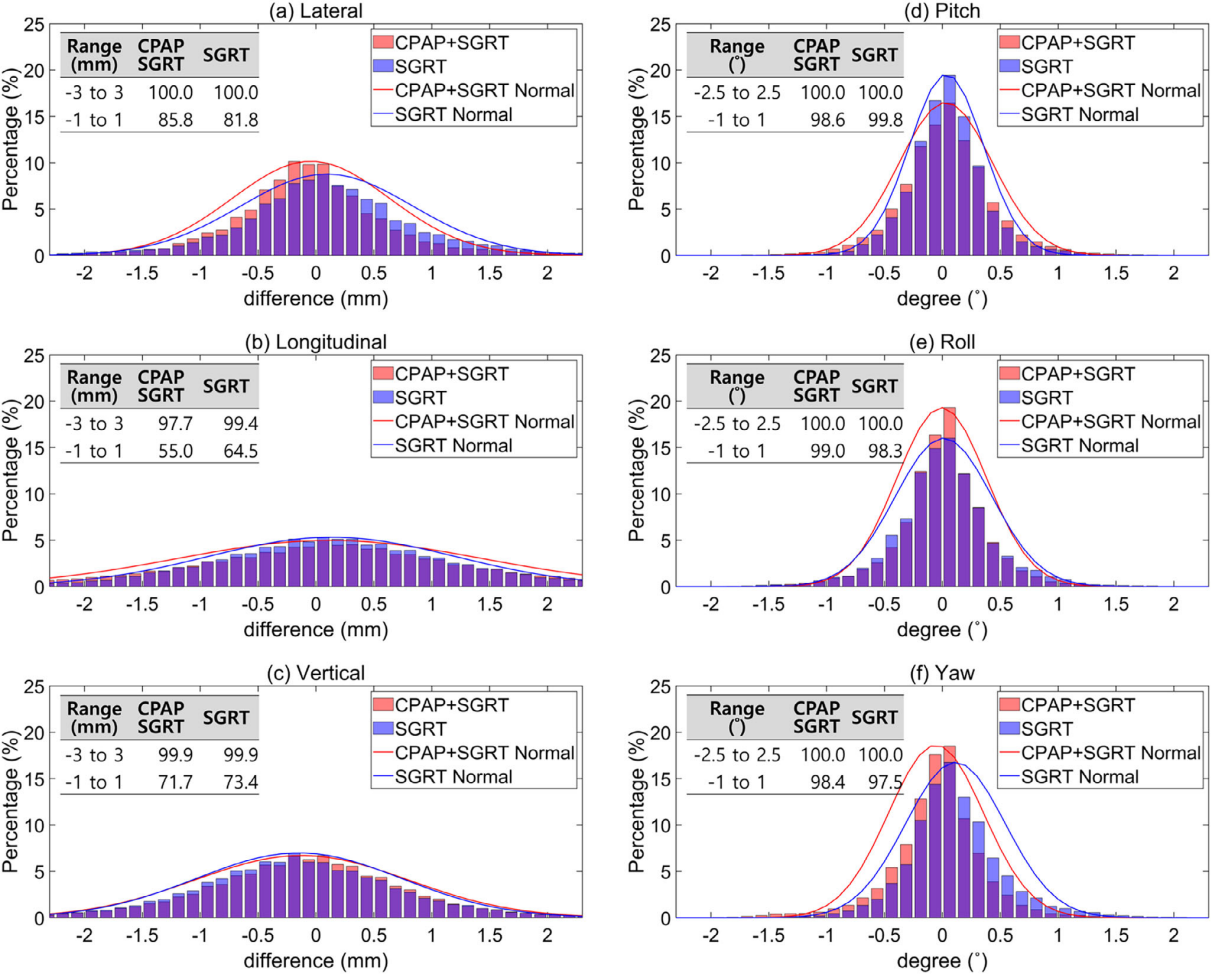
Normal distribution and histogram of six degrees of freedom (6DOF) displacement differences between live and reference images for the CPAP + SGRT and SGRT‐only groups. The data include three translational directions: (a) Lateral, (b) Longitudinal, (c) Vertical, and three rotational directions: (d) Pitch, (e) Roll, (f) Yaw. The percentages represent the proportion of measurements within specific displacement thresholds, with 97.7% or more within ± 3 mm (translation) and ± 2.5° (rotation) for both groups.

The displacement differences between live and reference images for the CPAP + SGRT and SGRT‐only groups were evaluated across the six degrees of freedom, as shown in Figure 2. In the translational directions (lateral, longitudinal, and vertical), both groups demonstrated high precision, with over 97% of measurements falling within ± 3 mm and a slightly lower percentage within ± 1 mm. Specifically, for the lateral axis, 85.8% of the CPAP + SGRT and 81.8% of the SGRT measurements were within 1 mm, whereas both groups achieved 100% within 3 mm. Similar trends were observed for the longitudinal and vertical directions, with the SGRT‐only group showing a slightly higher precision within ± 1 mm in the longitudinal axis.

For the rotational directions (pitch, roll, and yaw), both groups showed nearly identical performance, with over 98% of measurements within ± 1° and 100% within ± 2.5°. The CPAP + SGRT group exhibited slightly better precision in the yaw axis within ± 1°, achieving 98.4% compared to 97.5% for the SGRT‐only group. Overall, both groups demonstrated comparable accuracies across all axes, achieving excellent alignment within clinically acceptable tolerances.

**FIGURE 3:**
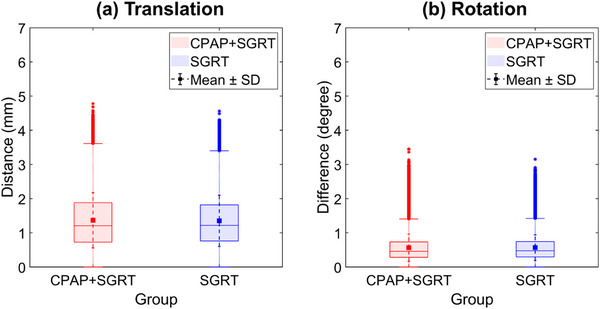
Box plots of translation (left) and rotation (right) vectors during beam‐on periods within gating thresholds (3 mm, 2.5) for CPAP + SGRT and SGRT groups. Mean ± SD is indicated by small square markers and dotted error bars.

To assess the gating effectiveness, displacement data confined within the preset thresholds (3 mm translation and 2.5° rotation) were separately analyzed. As shown in Figure 3 and Table 2, the mean translational and rotational displacements during beam‐on periods were 1.37 ± 0.81 mm and 0.57 ± 0.40° in the CPAP + SGRT group, and 1.35 ± 0.75 mm and 0.57 ± 0.38° in the SGRT group, respectively. These findings suggest that the patients were well stabilized during gated beam delivery, with no significant difference between the two groups.

**TABLE 2:** Summary of translation and rotation magnitudes during beam‐on intervals within gating thresholds, averaged across all treatment fractions. No significant differences were observed between groups (bootstrap *p* > 0.5). Translation values are presented in millimeters (mm), and rotation values are presented in degrees (°).

Displacement	Group	Mean	Median	Std	Max	Min	Bootstrap *p*‐value
**Translation**	**CPAP + SGRT**	1.37	1.21	0.81	4.78	0.02	0.52
	**SGRT**	1.35	1.22	0.75	4.56	0.01	
**Rotation**	**CPAP + SGRT**	0.57	0.46	0.40	3.45	0.00	0.53
	**SGRT**	0.57	0.48	0.38	3.15	0.00	

### 3.2 Treatment time metrics and gating efficiency

**FIGURE 4:**
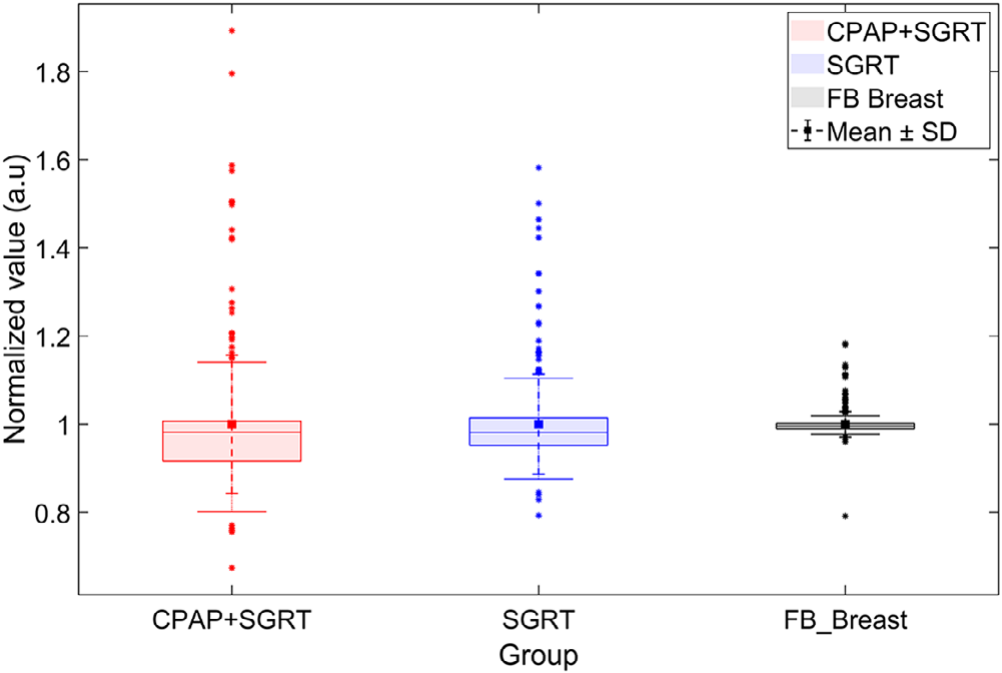
Treatment delivery time distribution normalized to each mean for both SGRT groups and the free‐breathing (FB Breast) group. The FB Breast group displays a narrower distribution compared to the two SGRT groups.

The variability in treatment delivery time due to beam hold activation was analyzed and compared across the CPAP + SGRT, SGRT‐only, and FB groups. The normalized treatment delivery time Std was 0.16 for the CPAP + SGRT group, 0.11 for the SGRT‐only group, and 0.03 for the FB group (Figure 4, Table 3).

**TABLE 3:** To analyze the distribution of normalized treatment delivery time for both SGRT groups and the FB Breast group, Std was calculated, and a Mann–Whitney U test was performed. Normalized values are unitless and were obtained by dividing the treatment delivery time of each fraction by the mean treatment delivery time of the corresponding patient.

Group	Median	Std	*p*‐value
**CPAP + SGRT**	0.982	0.16	
**SGRT**	0.981	0.11	0.408
**FB breast**	0.996	0.03	0.001

Mann–Whitney U test revealed a significant difference in the distribution of treatment delivery time between the SGRT groups, comprising CAPA + SGRT and SGRT‐only, and FB groups (*p* < 0.01). The broader distribution observed in the SGRT groups indicated increased variability in treatment delivery time compared with the FB group (Table 2). This suggests that the real‐time monitoring and beam‐hold activations employed in SGRT, while enhancing treatment precision, contributed to longer and more variable treatment delivery time compared with conventional FB radiation therapy.

**FIGURE 5:**
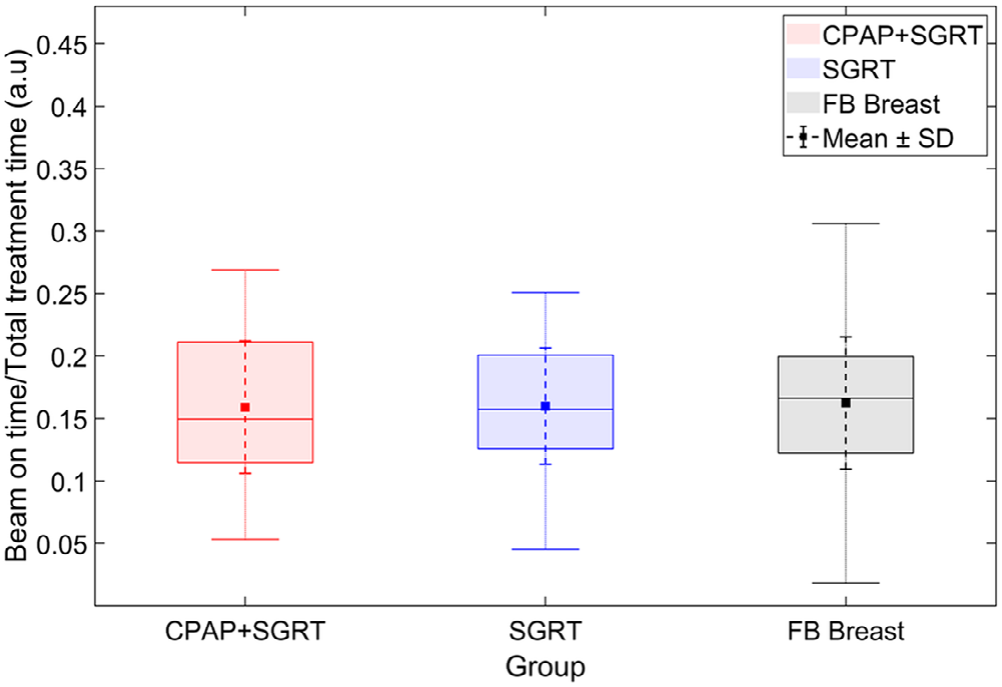
Beam‐on time normalized to total treatment time for the CPAP + SGRT, SGRT‐only, and FB Breast groups. Group distributions are shown as box plots, and Std is indicated by small dotted error bars.

To further assess treatment delivery efficiency beyond normalized time variability, we evaluated the duty cycle, defined as the ratio of beam‐on time (i.e., the time during which radiation was delivered within the gating threshold) to the total treatment time. Statistical analysis of the duty cycle values was performed using the Mann–Whitney U test. As shown in Figure 5 and Table 4, the mean duty cycle was 0.159 for the CPAP + SGRT group, 0.160 for the SGRT‐only group, and 0.162 for the FB group, with no statistically significant differences observed (CPAP + SGRT vs. SGRT: *p* = 0.610; CPAP + SGRT vs. FB: *p* = 0.604). These findings suggest that the use of CPAP does not adversely impact beam‐on efficiency and can be implemented without compromising treatment workflow. The normalized beam‐on time reflects temporal variability during radiation beam delivery, whereas the duty cycle specifically quantifies efficiency during gated beam‐on periods.

**TABLE 4:** The duty cycle—defined as the ratio of beam‐on time to total treatment time—was compared among the CPAP + SGRT, SGRT‐only, and FB Breast groups. The mean and Std were calculated, and a Mann–Whitney U test was performed. As a ratio of time values, the duty cycle is a unitless quantity.

Group	Mean	Std	P‐value
CPAP + SGRT	0.159	0.053	
SGRT	0.160	0.047	0.610
FB breast	0.162	0.053	0.604

**FIGURE 6:**
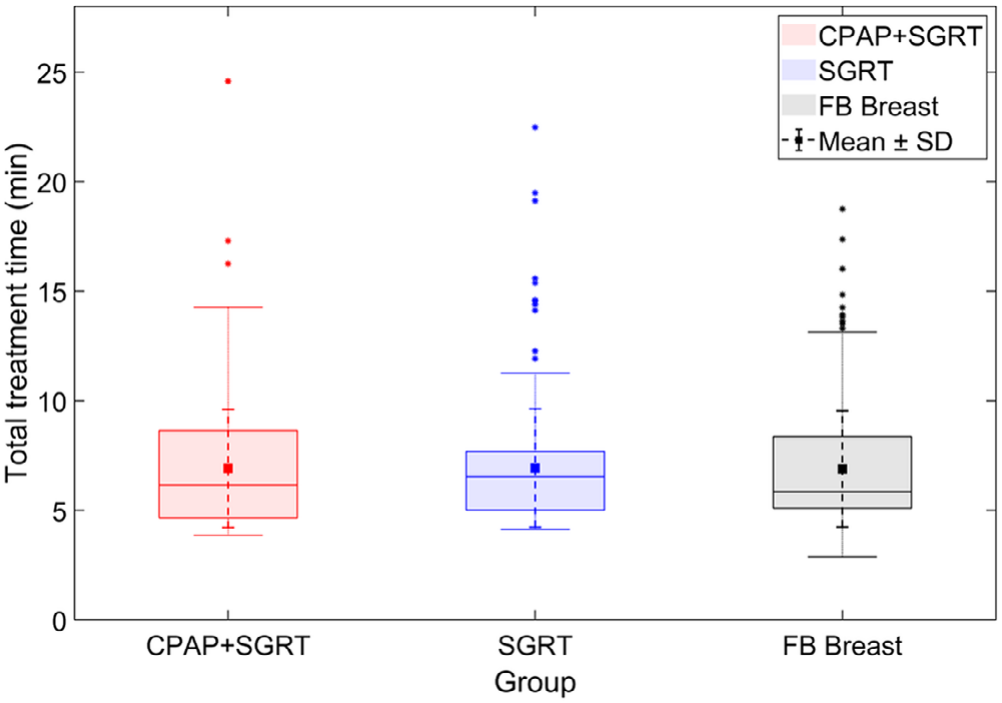
Box plots of total treatment time for the CPAP + SGRT, SGRT‐only, and FB Breast groups. Mean and Std are indicated by squares and small dotted error bars, respectively. The distributions illustrate the variation in total treatment time across the three groups.

In addition to analyzing normalized treatment delivery time variability and beam‐on efficiency, we evaluated the total treatment time to assess the potential impact of each technique on clinical workflow. As shown in Table 5 and Figure 6, the mean total treatment time was 6.914 ± 2.686 min for the CPAP + SGRT group, 6.936 ± 2.695 min for the SGRT‐only group, and 6.892 ± 2.643 min for the FB Breast group. Statistical comparison using the Mann–Whitney U test revealed no significant differences between groups (*p* > 0.5). These findings indicate that the incorporation of CPAP does not increase the total treatment time, thereby supporting its integration into routine clinical workflows without additional burden.

**TABLE 5:** Total treatment time (min) for the CPAP + SGRT, SGRT‐only, and FB Breast groups. Mean and Std were calculated, and group differences were assessed using the Mann–Whitney U test.

Group	Mean (min)	Std (min)	P‐value
CPAP + SGRT	6.914	2.686	
SGRT	6.936	2.695	0.562
FB breast	6.892	2.643	0.874

## 4 DISCUSSION

This study evaluated the impact of CPAP on breast positional reproducibility and treatment time metrics in SGRT, comparing CPAP + SGRT and SGRT‐only groups, as well as SGRT groups to an FB cohort. These findings highlighted the potential benefits and challenges of integrating SGRT with CPAP in clinical practice.

The positional reproducibility achieved with CPAP + SGRT was comparable to that achieved with SGRT alone. As shown in Figure 1 and Table 1, the analysis of patient displacement revealed no statistically significant differences in translation and rotation vectors between the CPAP + SGRT and SGRT‐only groups.

The displacement histograms, as shown in Figure 2, demonstrated that over 97% of measurements in both groups were within ± 3 mm, and 98% were within ± 1°, indicating high positional accuracy. The majority of patient displacement for both groups was contained within ± 3 mm and ± 2.5°. These findings validate effectiveness of SGRT as a stand‐alone tool for minimizing patient displacement during treatment, whereas CPAP provides complementary internal stabilization that enhances treatment accuracy in specific clinical scenarios. However, as highlighted in Table 1, instances of significant displacement were observed, with maximum translation vectors reaching 10.98 mm and rotation vectors reaching 5.41°. These outliers emphasize the critical importance of real‐time monitoring. The automatic beam‐hold feature of SGRT, which pauses radiation delivery when the displacement exceeds predefined thresholds (3 mm for translation and 2.5° for rotation), ensures precise radiation delivery. Without this safeguard, significant deviations could compromise dose accuracy and increase radiation exposure to surrounding normal tissues.^22^

To better understand displacement control under clinically acceptable limits, we separately analyzed patient displacement occurring within the gating thresholds (3 mm for translation and 2.5° for rotation) during beam‐on periods. The mean translation and rotation vectors during beam‐on periods were 1.37 ± 0.81 mm and 0.57 ± 0.40° for the CPAP + SGRT group, and 1.35 ± 0.75 mm and 0.57 ± 0.38° for the SGRT group, respectively. These values fall well within the threshold recommendations of AAPM TG‐302 for DIBH techniques (3–5 mm translation, 2–3° rotation), suggesting effective displacement control during gated radiation delivery.^1^

CPAP contributes an additional advantage by stabilizing internal anatomy—a benefit that may not be fully evident in external surface displacement measurements. Although CPAP is known to maintain lung inflation and reduce internal organ motion, these effects are not always directly captured by surface monitoring systems.^11^ The displacement observed in the CPAP + SGRT group likely reflects a complex interplay between altered respiratory mechanics under positive pressure and changes in surface displacement, such as subtle chest wall or diaphragmatic shifts, rather than a failure of internal stabilization.^23^ Moreover, continuous airflow from CPAP may induce subtle chest wall motion, which can be detected by sensitive SGRT monitoring systems even when internal structures remain stable.^23^ Previous studies by Jacobson et al. and Choi et al. demonstrated that CPAP significantly reduced radiation exposure to critical organs, such as the heart and lungs, particularly in patients with left‐sided breast cancer.^5^, ^6^ These findings align with those of multiple studies on deep inspiration breath‐hold (DIBH) techniques incorporating CPAP.^23^, ^24^ The synergy between external displacement control via SGRT and internal stabilization through CPAP demonstrates the clinical relevance of combining these techniques, particularly in thoracic radiation therapy, in which minor organ displacement can significantly impact treatment precision.^2^, ^5^, ^6^, ^25^ These results highlight the potential clinical advantages of combining SGRT with CPAP therapy.

Analysis of the treatment delivery time revealed an expected trade‐off between precision and efficiency. The normalized Std of treatment delivery time was 0.16 in the CPAP + SGRT group, 0.11 in the SGRT‐only group, and 0.03 in the FB group. The broader distribution of treatment delivery time in the SGRT group reflects the frequent beam‐hold activations triggered when the displacement thresholds were exceeded. Although these interruptions extend the treatment time, they are essential for maintaining precision by preventing radiation delivery during significant displacement.

This real‐time gating feature of SGRT enables more precise and stable treatment, particularly for patients with unpredictable motion patterns, by reducing the risk of geographic misses and limiting radiation exposure to surrounding organs, as also reported by Freislederer et al.^2^ CPAP may further contribute to internal anatomical stabilization, potentially reducing the frequency and magnitude of displacement‐induced interruptions.^26^

While the increased treatment delivery time variability is a necessary compromise to ensure accuracy, our findings indicate that this does not translate into a clinically meaningful extension of total treatment time. Specifically, duty cycle values remained comparable among all groups, and no statistically significant differences were observed in total treatment time. These results support the practical integration of SGRT and CPAP into routine workflows without compromising efficiency.

Future studies should investigate workflow optimization strategies, such as predictive motion modeling or adaptive thresholding, to minimize beam‐on interruptions and enhance treatment efficiency while preserving accuracy. Such developments could help further enhance the balance between precision and efficiency in SGRT‐guided breast cancer radiation therapy.

## 5 CONCLUSION

This study demonstrates that SGRT, with or without CPAP, provides effective surface displacement management during breast cancer radiation therapy, supporting precise and reproducible dose delivery. SGRT enables real‐time surface monitoring, while CPAP offers additional internal anatomical stabilization, particularly in patients prone to respiration‐induced surface displacement. Although SGRT was associated with increased variability in treatment delivery time due to gating, duty cycle, and total treatment time remained comparable across groups, indicating minimal impact on clinical workflow efficiency. These findings support the clinical feasibility of integrating SGRT and CPAP to enhance treatment precision without compromising throughput.

## AUTHOR CONTRIBUTION

The authors confirm their contribution to the paper as follows: **Jin Dong Cho**: Conceived the study, designed the methodology, performed data curation, formal analysis, investigation, and visualization, wrote the original draft, and contributed to reviewing and editing the manuscript. **Su Chul Han**: Contributed to the conceptualization and validation of the study, and participated in reviewing and editing the manuscript. **Heerim Nam**: Supervised the study and contributed to reviewing and editing the manuscript. **Jason Joon Bock Lee and Hyebin Lee**: Contributed to the critical review and editing of the manuscript. All authors reviewed the results and approved the final version of the manuscript.

## CONFLICT OF INTEREST STATEMENT

The authors have no relevant conflicts of interest to disclose.

## References

[1] HA Al‐Hallaq , Cerviño L , Gutierrez AN , et al. AAPM task group report 302: surface‐guided radiotherapy. Med Phys. 2022;49(4):e82‐e112. doi:10.1002/mp.15532 35179229 PMC9314008

[2] Freislederer P , Kügele M , Öllers M , et al. Recent advanced in surface guided radiation therapy. Radiat Oncol. 2020;15(1):1‐11. doi:10.1186/s13014-020-01629-w PMC7393906 32736570

[3] Chow VUY , Cheung MLM , Kan MWK , Chan ATC , Clinical experience of intrafractional motion monitoring of patients under head and neck radiation therapy using exactrac dynamic system. Adv Radiat Oncol. 2024;9(3):101390. doi:10.1016/j.adro.2023.101390 38292891 PMC10823086

[4] Darréon J , Massabeau C , Geffroy C , Maroun P , Simon L , Surface‐guided radiotherapy overview: technical aspects and clinical applications. Cancer Radiother. 2023;27(6‐7):504‐510. doi:10.1016/j.canrad.2023.07.003 37558608

[5] Jacobson G , Lawrence YR , Appel S , et al. Benefits of continuous positive airway pressure (CPAP) during radiation therapy: a prospective trial. Int J Radiat Oncol Biol Phys. 2021;110(5):1466‐1472. doi:10.1016/j.ijrobp.2021.03.044 33965269

[6] Choi MS , Park RH , Lee J , et al. Dosimetric comparison of CPAP and DIBH for left‐sided breast cancer radiation therapy. Adv Radiat Oncol. 2024;9(6):101478. doi:10.1016/j.adro.2024.101478 38681894 PMC11043855

[7] Jung I , Ha J , Chang W , et al. The efficacy of continuous positive airway pressure (CPAP) for patient with left breast cancer. J Korean Soc Radiat Ther. 2019;31(2):43‐49. https://www.koreascience.or.kr/article/JAKO201912062688469.page

[8] Kil WJ , Pham T , Hossain S , Casaigne J , Jones K , Khalil M , The impact of continuous positive airway pressure on radiation dose to heart and lung during left‐sided postmastectomy radiotherapy when deep inspiration breath hold technique is not applicable: a case report. Radiat Oncol J. 2018;36(1):79‐84. doi:10.3857/roj.2018.00017 29506325 PMC5903364

[9] Bin ParkJ , JH Lee , Chang JH , et al. Optimizing target and diaphragmatic configuration, and dosimetric benefits using continuous positive airway pressure in stereotactic ablative radiotherapy for lung tumors. Radiat Oncol J. 2024;42(3):200‐209. doi:10.3857/roj.2024.00101 39354823 PMC11467486

[10] Goodall SK , Rampant PL , Initial end‐to‐end testing of the ExacTrac dynamic deep inspiration breath hold workflow using a breath hold breast phantom. Phys Eng Sci Med. 2023;46(3):1239‐1247. doi:10.1007/s13246-023-01291-y 37349630 PMC10480281

[11] Allen AM , Ceder YK , Shochat T , et al. CPAP (Continuous Positive Airway Pressure) is an effective and stable solution for heart sparing radiotherapy of left sided breast cancer. Radiat Oncol. 2020;15(1):59. doi:10.1186/s13014-020-01505-7 32143658 PMC7060550

[12] Chow VUY , Cheung MLM , Kan MWK , Chan ATC , Shift detection discrepancy between ExacTrac Dynamic system and cone‐beam computed tomography. J Appl Clin Med Phys. 2022;23(5):1‐7. doi:10.1002/acm2.13567 PMC9121052 35188333

[13] Stanley DN , McConnell KA , Kirby N , Gutiérrez AN , Papanikolaou N , Rasmussen K , Comparison of initial patient setup accuracy between surface imaging and three point localization: a retrospective analysis. J Appl Clin Med Phys. 2017;18(6):58‐61. doi:10.1002/acm2.12183 28901684 PMC5689923

[14] Yaghoubi V , Vakilzadeh MK , Abrahamsson TJS , Automated modal parameter estimation using correlation analysis and bootstrap sampling. Mech Syst Signal Process. 2018;100:289‐310. doi:10.1016/j.ymssp.2017.07.004

[15] Perišić S , Barle J , Tomac I , Đukić P , Automatic damping estimation via bootstrap technique and Bayesian analysis for mechanical system condition monitoring. Mech Syst Signal Process. 2024;220:111654. doi:10.1016/j.ymssp.2024.111654

[16] Zoubir A , Iskandler D , Bootstrap methods and applications. IEEE Signal Process Mag. 2007;24(4):10‐19. doi:10.1109/MSP.2007.4286560

[17] Bańka P , Lurka A , Szuła Ł , Ground motion prediction of high‐energy mining seismic events: a bootstrap approach. Energies, 2023;16(10):4075. doi:10.3390/EN16104075

[18] Nachar N , The Mann‐Whitney U: a test for assessing whether two independent samples come from the same distribution. Tutor Quant Methods Psychol. 2008;4(1):13‐20. doi:10.20982/tqmp.04.1.p013

[19] Okeh UM , Statistical analysis of the application of Wilcoxon and Mann‐Whitney U test in medical research studies. Biotechnol Mol Biol Rev. 2009;4(6):128‐131. http://www.academicjournals.org/bmbr

[20] Rosner B , Grove D , Use of the Mann‐WhitneyU‐test for clustered data. Stat Med. 1999;18(11):1387‐1400. doi:10.1002/(SICI)1097‐0258(19990615)18:11<1387::AID‐SIM126>3.0.CO;2‐V 10399203 10.1002/(sici)1097-0258(19990615)18:11<1387::aid-sim126>3.0.co;2-v

[21] Thabet AAK , Al‐Bahlooli SH , Al‐Kohlani A , Shoja'a A , Oseltamivir‐resistant pandemic (H1N1)2009 in Yemen—case report. Virol J. 2010;7(1):88. doi:10.1186/1743‐422X‐7‐88 20459681 10.1186/1743-422X-7-88 PMC2874539

[22] Foster RD , Moeller BJ , Robinson M , et al. Dosimetric analysis of intra‐fraction motion detected by surface‐guided radiation therapy during linac stereotactic radiosurgery. Adv Radiat Oncol. 2023;8(3):101151. doi:10.1016/j.adro.2022.101151 36691448 10.1016/j.adro.2022.101151 PMC9860342

[23] Liang E , Dolan JL , Morris ED , et al. Application of continuous positive airway pressure for thoracic respiratory motion management: an assessment in a magnetic resonance imaging–guided radiation therapy environment. Adv Radiat Oncol. 2022;7(3):100889. doi:10.1016/j.adro.2021.100889 35198838 10.1016/j.adro.2021.100889 PMC8844850

[24] Reckhow J , Kaidar‐Person O , MA Ben‐David , et al. Continuous positive airway pressure with deep inspiration breath hold in left‐sided breast radiation therapy. Med Dosim. 2021;46(2):127‐131. doi:10.1016/j.meddos.2020.09.006 33020023 10.1016/j.meddos.2020.09.006

[25] MacFarlane MJ , Jiang K , Mundis M , et al. Comparison of the dosimetric accuracy of proton breast treatment plans delivered with SGRT and CBCT setups. J Appl Clin Med Phys. 2021;22(9):153‐158. doi:10.1002/acm2.13357 34288378 10.1002/acm2.13357 PMC8425866

[26] Goldstein JD , Lawrence YR , Appel S , et al. Continuous positive airway pressure for motion management in stereotactic body radiation therapy to the lung: a controlled pilot study. Int J Radiat Oncol Biol Phys. 2015;93(2):391‐399. doi:10.1016/J.IJROBP.2015.06.011 26264628 10.1016/j.ijrobp.2015.06.011

